# Associations of one-carbon metabolism, related B-vitamins and *ApoE* genotype with cognitive function in older adults: identification of a novel gene-nutrient interaction

**DOI:** 10.1186/s12916-025-04276-8

**Published:** 2025-07-28

**Authors:** Shane Gordon, Leane Hoey, Helene McNulty, Jordan Keenan, Faith Pangilinan, Lawrence C. Brody, Mary Ward, J. J. Strain, Liadhan McAnena, Adrian McCann, Anne M. Molloy, Conal Cunningham, Kevin McCarroll, Catherine F. Hughes

**Affiliations:** 1 Nutrition Innovation Centre for Food & Health (NICHE), School of Biomedical Sciences, Ulster University, Coleraine, Northern Ireland BT52 1SA UK; 2 Genetics and Environment Interaction Section, National Human Genome Research Institute, National Institutes of Health, Bethesda, MD USA; 3 Bevital AS, Bergen, Norway; 4 School of Medicine, Trinity College Dublin, Dublin, Ireland; 5 Mercer’s Institute for Successful Ageing, St James’s Hospital, Dublin, Ireland; 6 Department of Medical Gerontology, School of Medicine, Trinity College Dublin, Dublin, Ireland

**Keywords:** Cognitive function, Folate, Vitamin B12, Vitamin B6, Riboflavin, Homocysteine, *Apolipoprotein E* (*ApoE* ε4), One-carbon metabolism, Trinity-Ulster-Department of Agriculture (TUDA)

## Abstract

**Background:**

The role of one-carbon metabolism and related B-vitamins in cognitive function in ageing is well-documented, particularly for folate and vitamin B12, with vitamin B6 and riboflavin receiving much less attention. *ApoE* is a well-established genetic risk factor for Alzheimer’s disease, but the role of B-vitamins in modifying this risk remains largely unexplored. We examined associations between folate, B12, B6, riboflavin, and cognitive function in older adults, including interactions with the *ApoE* ε4 genotype.

**Methods:**

Community-dwelling older adults (≥ 60 years) from the Trinity-Ulster-Department of Agriculture (TUDA) study were stratified as *ApoE* ε4 carriers (ε3/ε4 and ε4/ε4 genotypes; *n* = 1205) or non-ε4 carriers (*n* = 3348). Cognitive function was assessed using the Repeatable Battery for Assessment of Neuropsychological Status (RBANS), the Frontal Assessment Battery, and the Mini-Mental State Examination. Logistic regression models were used to evaluate the association between cognitive dysfunction (defined as RBANS score < 80) and a range of variables, including biomarkers of folate, vitamins B12, B6, and riboflavin status, plasma homocysteine levels, and *ApoE* ε4 genotype.

**Results:**

Lower status of vitamin B12 (holotranscobalamin; adjusted odds ratio (OR_adj_ 1.30; 95% CI: 1.08–1.58, *p* = 0.007), vitamin B6 (OR_adj_ 1.37; 95% CI: 1.12–1.67, *p* = 0.002), riboflavin (OR_adj_ 1.73; 95% CI: 1.44–2.09, *p* < 0.001), and higher plasma homocysteine (OR_adj_ 1.50; 95% CI: 1.22–1.83, *p* < 0.001) were each associated with higher risk of cognitive dysfunction. The *ApoE* ε4 genotype interacted adversely with low B12 status (*p* = 0.030) and elevated homocysteine (*p* = 0.008) in relation to cognitive outcomes.

**Conclusions:**

Low status of vitamin B12, B6, riboflavin, and/or elevated homocysteine were each associated with a greater risk of cognitive dysfunction. A novel interaction between *ApoE* ε4 and low B12 or higher homocysteine was associated with an increased risk of cognitive dysfunction. Improving B-vitamin status may have important public health benefits for dementia prevention. Further investigation, ideally in the form of randomised trials, is however required to demonstrate a causative relationship and confirm whether intervention with B-vitamins can confer a beneficial effect in maintaining better cognitive health in at-risk older people.

**Trial registration:**

TUDA study: ClinicalTrials.gov no. NCT02664584 (27/01/2016).

**Supplementary Information:**

The online version contains supplementary material available at 10.1186/s12916-025-04276-8.

## Background

Dementia is a complex multifactorial condition that results from the interaction of a variety of modifiable and non-modifiable factors [1, 2]. Although there is no cure for dementia, there have been significant advancements in disease-modifying treatments; results to date, however, have been mixed [1]. Identifying risk factors and understanding their potential interactions continue to be crucial for effective dementia prevention [3]. Genetic factors are known to play a role in the development of dementia, but only a small number of directly causative genetic variants have been identified [4]. These variants, which are typically associated with early-onset Alzheimer’s disease (AD) or frontotemporal dementia, account for less than 1% of cases [5], but individuals carrying these variants are recognised to have a lifetime risk of developing dementia exceeding 95% [4].

At the population level, a genetic mutation of the *apolipoprotein E* (*ApoE*) gene is the strongest genetic risk factor for AD [6], the most common form of dementia. The *ApoE* gene, located on chromosome 19, has three isoforms, ε2, ε3, and ε4, that influence the function of the ApoE cholesterol transporter [7]. Approximately 14% of the global population—rising to around 25% in Northern Europe—carry a single copy of the ε4 allele, whereas around 2% carry two copies [8–10]. Notably, carrying at least one ε4 allele is associated with a threefold increased risk of AD, and up to 12-fold in homozygotes (ε4/ε4), compared to the ε3/ε3 genotype [11]. Although 40–65% of people living with AD do not have the ε4 genotype [5], not all ε4 carriers develop AD, suggesting that nutrition and healthier lifestyle factors may help mitigate this genetic risk [12].

Nutrients such as omega-3 fatty acids, vitamin D, and B-vitamins have each been associated with a reduced risk of dementia [13, 14]. In particular, a substantial body of research indicates that folate and related B-vitamins involved in one-carbon metabolism may help promote better cognitive health in ageing [2, 15, 16]. One recent meta-analysis of 25 randomised controlled trials (RCTs) reported that B-vitamin supplementation resulted in a small but significant improvement in cognitive function, particularly in populations without dementia and when the intervention lasted longer than 12 months [17]. The most robust evidence comes from the VITACOG trial, which showed that 2 years of combined B-vitamin supplementation lowered homocysteine concentrations, slowed cognitive decline, and most importantly, reduced brain atrophy by 40% in individuals with mild cognitive impairment, as measured by MRI scans [18–20]. However, the majority of studies to date investigating the role of B-vitamins on cognitive health have not considered the impact of the *ApoE* ε4 genotype. Limited evidence indicates that the *ApoE* ε4 phenotype may be influenced by B-vitamin status. One such study conducted in older Chinese adults reported that vitamin B12 was positively associated with Mini-Mental State Examination (MMSE) scores and that this association was more pronounced in *ApoE* ε4 carriers [21]. This study, however, relied solely on serum total vitamin B12. Assessing B12 status using a single biomarker is not recommended; combining a direct marker (e.g. total B12 or holotranscobalamin) with a functional marker (e.g. homocysteine or methylmalonic acid) is preferred [22]. Furthermore, the MMSE is a cognitive screening tool only and is often criticised for its lack of sensitivity and ‘ceiling’ effects [23]. Nevertheless, another study in older adults reported that *ApoE* ε4 carriers with low vitamin B12 concentrations had impaired recognition of ‘dated famous faces’, but not short-term memory or visuospatial abilities [24]. Further evidence from brain imaging studies suggests that *ApoE* ε4 status may modify the effects of vitamin B12 and homocysteine on grey matter volumes. In a Korean cohort, a positive correlation was observed between serum vitamin B12 concentrations and grey matter volume in *ApoE* ε4 carriers with AD, whereas homocysteine was negatively correlated with grey matter volume in non-carriers [25].

To date, no study has examined the roles of the *ApoE* ε4 genotype and all the relevant B-vitamins involved in one-carbon metabolism in relation to cognitive health. Therefore, the objective of this study was to investigate the associations of folate, B12, B6, and riboflavin with cognitive function in older adults and to determine the interaction, if any, between the *ApoE* ε4 genotype and low status of one or more B-vitamins in determining cognitive outcomes.

## Methods

### Study cohort

This study utilised data from the Trinity-Ulster-Department of Agriculture (TUDA) cohort, a large, all-Ireland, population-based study that explores the impact of nutritional, genetic, and lifestyle factors on age-related diseases, with a specific focus on cardiovascular disease, osteoporosis, and dementia. As described in detail elsewhere [26], a total of 5186 adults aged ≥ 60 years were recruited between 2008 and 2012 from general practice surgeries and cardiology clinics in the Northern and Western Health and Social Care Trust in Northern Ireland (NI; *n* = 2100) and hospital outpatient clinics at St James Hospital Dublin, Republic of Ireland (RoI; *n* = 3086). The inclusion criteria were: age ≥ 60 years, born on the island of Ireland, and free from dementia at recruitment. Ethical approval was granted by the Office for Research Ethics Committees Northern Ireland (ORECNI; 26 March 2009; reference 08/NI/RO3113), with corresponding approvals from the Northern and Western Health and Social Care Trust and the Research Ethics Committee of St James Hospital and The Adelaide and Meath Hospital in Dublin (7 November 2008). All participants provided written informed consent at the time of recruitment. This study is reported as per the Strengthening the Reporting of Observational Studies in Epidemiology (STROBE) guidelines (Additional file 1: Table S1).

### Health and lifestyle measures

Anthropometric measurements were obtained using standardised techniques, and body mass index (BMI) was calculated as weight (kg) divided by height (m^2^). Functional assessments included the Instrumental Activities of Daily Living test, the Physical Self-Maintenance Scale, and the Timed Up-and-Go (TUG) frailty test. For the TUG test, participants rise from a standard-height chair, walk 3 m before turning around and return to the chair to sit down [27]. A time of ≥ 12 s to complete this test indicates frailty [28]. Detailed information on medical history, smoking, alcohol consumption, and prescription medication use was collected. A food frequency questionnaire was used to assess fortified food and supplement use. In accordance with clinical guidelines [29], two blood pressure measurements were taken from the reference arm, with a 5–10-min interval between each measurement to calculate a mean value. If the difference between the two measurements exceeded 5 mmHg, a third measurement was taken after 10–15 min. The mean of the two blood pressure measurements that were closest in value was used.

Socioeconomic deprivation was determined using a novel, geo-referenced, address-based system linked to deprivation indices. In NI, deprivation scores were based on income, employment, health, education, proximity to services, living environment, and crime. In the RoI, scores were based on demographic profile, social class composition, and labour market situation. Socioeconomic deprivation scores from both regions were combined into one standardised score and categorised into quintiles (quintile 1: least deprived; quintile 5: most deprived), as explained elsewhere [30].

### Neuropsychological tests

Neuropsychological performance was assessed by trained researchers using the Repeatable Battery for Assessment of Neuropsychological Status (RBANS), the Frontal Assessment Battery (FAB), and the MMSE. The RBANS is a comprehensive age-standardised tool that measures global cognitive function, along with five subdomains: *immediate memory*, which measures the ability to recall information immediately after presentation; *visuospatial/constructional skills*, which evaluate spatial perception and visual processing; *language*, which assesses naming abilities and verbal fluency; *attention*, which focuses on sustained and selective attention; and *delayed memory*, which evaluates the capacity to retain and retrieve information over time [31]. An RBANS total score < 80 indicates cognitive dysfunction. The MMSE is the most widely used clinical screening tool for assessing global cognitive function [32]. It has a maximum score of 30, with a score below 25 typically indicating cognitive dysfunction. Finally, the FAB is specifically designed to evaluate executive functioning associated with the frontal lobe by assessing six key domains: conceptualisation, verbal fluency, motor programming, resistance to interference, inhibitory control, and environmental autonomy [33]. The maximum score for the FAB is 18, with a score of 12 and below indicating cognitive dysfunction [34].

Anxiety and depression were measured using the 7-item Hospital Anxiety and Depression Scale [35] and the Center for Epidemiological Studies-Depression scale [36]. A cut-off score of ≥ 11 was used for anxiety, and a cut-off score of ≥ 16 was used for depression. Permission with relevant licenses for CES-D and HADS scales, as well as the neuropsychological scales (RBANS, MMSE and FAB), as used in this study, was obtained at the time of initial registration of the TUDA study. Additional self-reported diagnoses and medication/s used for anxiety and depression were also collected.

### Clinical and nutritional biomarkers

Non-fasting blood samples were obtained and analysed in hospital laboratories in Dublin and Northern Ireland for routine clinical health markers including a full blood count, renal and lipid profiles, glucose, and glycated haemoglobin (HbA1c). The samples for B-vitamin biomarker analysis were prepared and fractionated within 4 h of collection and were stored at −70 °C for batch analysis. Folate, vitamin B12, and homocysteine were analysed at Trinity College Dublin, whereas vitamin B6 and riboflavin were analysed at Ulster University, Coleraine. Serum and red blood cell (RBC) folate concentrations were determined via microbiological assays using *Lactobacillus leichmanni* [37]. Vitamin B12 status was assessed via total serum cobalamin concentrations, measured using a microbiological assay with *Lactobacillus delbrueckii*, whereas holotranscobalamin (holoTC) was measured by immunofluorescence [38]. Plasma pyridoxal 5′-phosphate (PLP; vitamin B6) was assessed using high-performance liquid chromatography [39]. Riboflavin was measured using the erythrocyte glutathione reductase activation coefficient (EGRac) before and after in vitro reactivation with flavin adenine dinucleotide, the active cofactor form of riboflavin [40]. The results are reported as a ratio; a lower EGRac ratio indicates a higher riboflavin status. Plasma total homocysteine was measured using a fluorescence polarisation immunoassay [41]. Lastly, serum 25-hydroxyvitamin D concentrations were quantified using liquid chromatography–tandem mass spectrometry.

### *ApoE* genotyping

DNA was extracted from the buffy coat, a component of whole blood, by Genuity Science (Dublin, Ireland), using an automated precipitation-based method on the Autogen FlexSTAR Plus. This system enables fully traceable and automated liquid handling for sample volumes ranging from 0.5 to 10 mL, with the DNA yield varying based on selected parameters. Up to 30 samples were processed simultaneously, with DNA eluted into a matrix tube compatible with both two-dimensional and one-dimensional barcode reading. Following extraction, quality control was performed using a high-throughput automated workflow on the Hamilton Microlab STAR. The DNA concentration was determined via spectrofluorometry using the quantitative PicoGreen Assay on the Varioskan Lux microplate reader. The samples were measured in triplicate against a standard curve, and the mean concentration was determined by the Hamilton system to normalise samples to 20 ng/µL for downstream applications.

Variant calling of single nucleotide polymorphisms (SNPs) was conducted by Genuity Science. *ApoE* genotypes were called from the Illumina Global Screening Array (*n* = 40) and the Axiom Precision Medicine Research Array (*n* = 4682), with a per-sample call rate of 97%. For 84 participants whose *ApoE* SNPs (rs7412 and rs429358) were not directly genotyped, imputation was performed using the Michigan Imputation Server with the 1000 Genomes reference panel. In total, 4676 individuals were successfully genotyped for rs7412 and rs429358, whose combinations define the three common *ApoE* alleles: ε2 (rs7412-T, rs429358-T), ε3 (rs7412-C, rs429358-T), and ε4 (rs7412-C, rs429358-C). The *ApoE* genotypes were grouped into non-ε4 carriers (*n* = 3348; ε2/ε2, ε2/ε3, and ε3/ε3) and ε4 carriers (*n* = 1205; ε3/ε4 and ε4/ε4). Participants with the *ApoE* ε2/ε4 genotype (*n* = 93) were excluded from further analysis, as this genotype contains both the protective ε2 allele and the risk ε4 allele [7], resulting in a final analytic sample of 4,553 TUDA participants.

### Statistical analysis

Data preparation was conducted using R (version 4.4.3), focusing on non-SNP variables, including winsorisation of extreme values (*DeskTools* package), assessment of missing values, and imputation of missing values using the *Multiple Imputation by Chained Equations* package to ensure data completeness before merging with the *ApoE* SNP data. Statistical analyses were then performed using the Statistical Package for the Social Sciences (version 29.0.1.0), with transformations applied where necessary to meet the assumption of normality.

Independent samples *t*-tests were performed to compare baseline characteristics by sex and those stratified by *ApoE* ε4 status, whereas chi-square tests were applied to categorical variables. Adjusted means were calculated and displayed for neuropsychological test scores, controlling for age, sex, education, socioeconomic deprivation (most deprived), obesity (waist circumference of ≥ 102 cm for males; ≥ 88 cm for females), LDL cholesterol, and depression.

Logistic regression models were used to assess associations between B-vitamin biomarkers (categorised into quartiles) and cognitive dysfunction (RBANS total score < 80), with quartile 1 (highest status) as the reference for each biomarker. Logistic regression models were also used to assess potential interactions between *ApoE* ε4 carrier status and low B-vitamin status (lower quartiles) to explore whether *ApoE* ε4 influences the relationship between B-vitamin status and cognitive dysfunction, adjusting for relevant covariates. *P*-values < 0.05 were considered statistically significant.

## Results

**Fig. 1:**
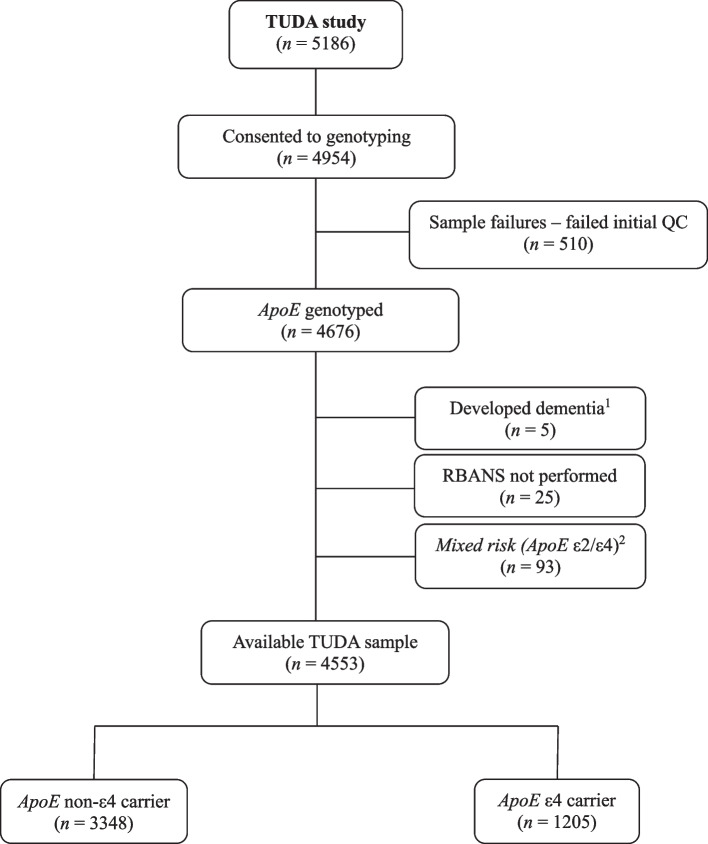
Flow diagram of study participants from the TUDA study. ^1^ Participants were excluded as a diagnosis of dementia was made since recruitment as per the TUDA study exclusion criteria. ^2^ The ε2/ε4 genotype was excluded from the analysis, as it contains both the protective ε2 allele and the risk ε4 allele [7]. Abbreviations: RBANS, Repeatable Battery for Neuropsychological Status; TUDA, Trinity-Ulster-Department of Agriculture

Among the total TUDA sample, 4553 participants genotyped for *ApoE* were selected for this analysis (Fig. 1). As shown in Table 1, females were significantly older than males, but there were no differences in education or socioeconomic deprivation. Male participants had significantly higher BMI, but a greater proportion of females were classified as living with obesity using sex-specific cut-offs (waist circumference of ≥ 102 cm for males; ≥ 88 cm for females). Males exhibited significantly higher blood pressure, triglycerides, HbA1c, and c-reactive protein, whereas females had significantly higher HDL and LDL cholesterol concentrations and had a significantly better B-vitamin status. No significant differences were observed between males and females for cognitive performance, as measured by MMSE and FAB, but females scored higher on the RBANS even after adjusting for relevant covariates. Also, females had higher rates of depression and anxiety scores. Overall, 25.8% of males and 24.1% of females were *ApoE* ε4 carriers.

**Table 1:** General characteristics of TUDA study participants

	**Males** **(*n* = 1440)**	**Females** **(*n* = 3113)**	***P***
Age, years	73.6 ± 8.0	74.5 ± 8.4	< 0.001
Education^a^, years	16.0 ± 3.2	16.0 ± 2.8	0.905
Socioeconomic deprivation, *n* (%)			
Quintile 1 (least deprived)	276 (19.2)	660 (21.2)	0.166
Quintile 5 (most deprived)	379 (26.3)	791 (25.4)	
Health and lifestyle factors			
Waist circumference (cm)	102.4 ± 12.4	91.8 ± 13.6	< 0.001
Obesity^b^, *n* (%)	718 (49.9)	1895 (60.9)	< 0.001
Body mass index, kg/m^2^	28.5 ± 4.5	27.6 ± 5.8	< 0.001
Timed Up-and-Go, seconds	14.0 ± 9.4	14.7 ± 9.2	< 0.001
PSMS total score	23.2 ± 1.7	22.9 ± 2.0	< 0.001
IADL total score	23.9 ± 4.2	24.2 ± 4.6	< 0.001
Clinical markers			
Systolic blood pressure, mmHg	147.3 ± 20.4	142.7 ± 21.2	< 0.001
Diastolic blood pressure, mmHg	79.4 ± 11.5	77.4 ± 11.0	< 0.001
Hypertension^c^, *n* (%)	1144 (80.4)	2090 (67.6)	< 0.001
Triglycerides, mmol/L	1.5 ± 0.8	1.4 ± 0.6	< 0.001
LDL cholesterol, mmol/L	2.2 ± 0.8	2.6 ± 0.9	< 0.001
HDL cholesterol, mmol/L	1.2 ± 0.3	1.5 ± 0.4	< 0.001
Glycated haemoglobin (HbA1c; mmol/mol)	41.3 ± 8.9	40.0 ± 7.0	< 0.001
C-reactive protein (CRP; mg/L)	3.2 ± 3.6	2.9 ± 3.0	0.017
Nutritional biomarkers			
Red blood cell folate, nmol/L	920 ± 480	977 ± 536	< 0.001
Serum folate, nmol/L	23.6 ± 15.9	28.6 ± 21.0	< 0.001
Total vitamin B12, pmol/L	239 ± 109	267 ± 127	< 0.001
Holotranscobalamin, pmol/L	53.1 ± 28.5	61.0 ± 35.9	< 0.001
Plasma pyridoxal 5′-phosphate (B6), nmol/L	58.7 ± 34.9	62.7 ± 41.8	< 0.001
EGRac (riboflavin)	1.34 ± 0.19	1.33 ± 0.19	0.047
Plasma total homocysteine, µmol/L	14.7 ± 4.7	13.7 ± 4.5	< 0.001
Serum 25-hydroxyvitamin D, nmol/L	52.5 ± 27.7	62.3 ± 32.4	< 0.001
Neuropsychological tests^d^			
MMSE total score	26.9 (26.8–27.1)	27.0 (27.0–27.1)	0.118
FAB total score	15.1 (15.0–15.2)	15.2 (15.2–15.3)	0.119
RBANS total score	84.2 (83.4–85.0)	85.8 (85.2–86.3)	0.002
Cognitive dysfunction, *n* (%)	268 (18.6)	618 (19.9)	0.345
Depression (CES-D total score)	3.0 ± 5.2	4.0 ± 6.3	< 0.001
Self-reported depression, *n* (%)	279 (19.4)	826 (26.5)	< 0.001
Anxiety (HADS total score)	1.5 ± 2.7	2.2 ± 3.3	< 0.001
Self-reported anxiety, *n* (%)	224 (15.6)	758 (24.3)	< 0.001
*ApoE* ε4 genotype			
Non ε4 carrier, *n* (%)	1030 (71.5)	2318 (74.5)	
ε4 carrier, *n* (%)	372 (25.8)	751 (24.1)	0.005
ε4/ε4 carrier, *n* (%)	38 (2.6)	44 (1.4)	

Data is presented as mean ± SD unless otherwise stated. *P*-values obtained from independent samples *t*-test for continuous variables and chi-squared analysis for categorical variables. *P* < 0.05 denotes statistical significance

*Abbreviations:* *CES-D* Center for Epidemiological Studies-Depression, *CI* confidence interval, *EGRac* erythrocyte glutathione reductase activation coefficient, *FAB* Frontal Assessment Battery, *HADS* Hospital Anxiety and Depression Scale, *IADL* Instrumental Activities of Daily Living, *MMSE* Mini-Mental State Examination, *RBANS* Repeatable Battery for Neuropsychological Status, *TUDA* Trinity-Ulster-Department of Agriculture

^a^Age formal education ended

^b^Waist circumference of ≥ 102 cm for males; ≥ 88 cm for females

^c^Systolic blood pressure ≥ 140 mmHg and/or diastolic blood pressure ≥ 90 mmHg)

^d^Displays adjusted means, 95% CIs; controlling for age, education, socioeconomic deprivation, obesity, LDL cholesterol, depression and *ApoE* status

Health and lifestyle determinants of cognitive dysfunction were examined in Table 2 using logistic regression. The risk of cognitive dysfunction increased with age and decreased with education; each additional year spent in formal education was associated with a 21% lower risk of cognitive dysfunction. Greater socioeconomic deprivation was associated with a 51% greater risk of cognitive dysfunction (adjusted odds ratio (OR_adj_ 1.51; 95% CI: 1.30–1.77; *p* < 0.001). Among all the determinants, *ApoE* ε4 carrier status was associated with the highest risk of cognitive dysfunction, with carriers being 56% more likely to experience cognitive dysfunction compared to non-carriers (OR_adj_ 1.56; 95% CI: 1.34–1.83; *p* < 0.001). Factors such as sex, living alone, hypertension, obesity, and anxiety were not significantly associated with cognitive dysfunction in this cohort.

**Table 2:** General health and lifestyle determinants of cognitive dysfunction

	***β***	**OR**_**adj**_	**95% CI**	***P***
Sociodemographic factors				
Age, years	0.070	1.07	1.06–1.08	< 0.001
Female sex	− 0.143	0.87	0.74–1.01	0.073
Education^a^, years	− 0.236	0.79	0.76–0.82	< 0.001
Socioeconomic deprivation^b^	0.414	1.51	1.30–1.77	< 0.001
Living alone	0.052	1.05	0.91–1.22	0.494
Health and lifestyle factors				
Visual impairment	0.401	1.49	1.12–1.99	0.007
Hypertensive^c^	− 0.006	0.99	0.85–1.17	0.944
Obesity (waist circumference)	− 0.045	0.96	0.83–1.11	0.551
LDL cholesterol, mmol/L	− 0.097	0.91	0.84–0.99	0.022
HbA1c, mmol/mol	0.008	1.01	1.00–1.02	0.046
Alcohol consumer (current)	− 0.219	0.80	0.70–0.93	0.002
Smoking status (current)	0.345	1.41	1.14–1.75	0.001
Physical inactivity^d^	0.316	1.37	1.17–1.61	< 0.001
Depression (CES-D total score)	0.037	1.04	1.03–1.05	< 0.001
Anxiety (HADS total score)	− 0.006	0.99	0.97–1.01	0.555
* ApoE* ε4 carrier^e^	0.447	1.56	1.34–1.83	< 0.001

*P*-values were obtained from logistic regression analysis of cognitive dysfunction (RBANS total score < 80). *P* < 0.05 denotes statistical significance

*Abbreviations:* *CES-D* Center for Epidemiological Studies-Depression, *CI* confidence interval, *HADS* Hospital Anxiety and Depression Scale, *HbA1c* glycated haemoglobin, *LDL* low-density lipoprotein, *OR*_*adj*_ adjusted odds ratio, *RBANS* Repeatable Battery for Neuropsychological Status

^a^Age formal education ended

^b^Most deprived quintile

^c^Systolic blood pressure ≥ 140 mmHg and/or diastolic blood pressure ≥ 90 mmHg)

^d^No exercise in previous 2 weeks

^e^*ApoE* ε4 carriers (ε3/ε4 and ε4/ε4)

Cognitive function scores, as well as general health and lifestyle characteristics of participants stratified by *ApoE* ε4 genotype, are shown in Table 3. *ApoE* ε4 carriers were significantly younger than non-carriers and had significantly higher LDL cholesterol concentrations. With regards to cognitive function, *ApoE* ε4 carriers had significantly lower total scores of MMSE, FAB and RBANS (RBANS score: 81.9, 95% CI: 81.0–82.7) compared with non ε4 carriers (85.7, 95% CI: 85.2–86.2) after adjusting for relevant covariates. In total, 42% of ε4 carriers were classified as having cognitive dysfunction, compared with 35% of non-carriers.

**Table 3:** Comparison of clinical, metabolic, and cognitive characteristics by *ApoE* genotype

	***ApoE* non-ε4 carrier** **(*n* = 3348)**	***ApoE* ε4 carrier**^**a**^ **(*n* = 1205)**	***P***
Age, years	74.4 ± 8.4	73.7 ± 7.9	0.009
Female sex, *n* (%)	2318 (69.2)	795 (66.0)	0.040
Education^b^, years	16.0 ± 2.9	16.1 ± 2.9	0.507
Health and lifestyle factors			
Waist circumference, cm	95.4 ± 14.0	94.4 ± 14.2	0.033
Obesity^c^, *n* (%)	1969 (58.8)	644 (53.4)	< 0.001
Timed-Up-and-Go, seconds	14.6 ± 9.3	14.2 ± 9.1	0.200
Clinical markers			
Triglycerides, mmol/L	1.4 ± 0.7	1.4 ± 0.7	0.107
LDL cholesterol, mmol/L	2.4 ± 0.9	2.5 ± 0.9	< 0.001
HDL cholesterol, mmol/L	1.4 ± 0.4	1.4 ± 0.4	0.414
B-vitamin biomarkers			
Red blood cell folate, nmol/L	955 ± 520	967 ± 516	0.473
Serum folate, nmol/L	26.6 ± 19.2	27.7 ± 20.0	0.095
Total vitamin B12, pmol/L	256 ± 121	261 ± 124	0.982
Holotranscobalamin, pmol/L	58.4 ± 33.3	58.4 ± 34.5	0.247
PLP (vitamin B6), nmol/L	61.6 ± 39.2	60.9 ± 40.8	0.568
EGRac (riboflavin)	1.33 ± 0.19	1.33 ± 0.19	0.348
Plasma homocysteine, µmol/L	14.0 ± 4.6	13.8 ± 4.6	0.248
Neuropsychological tests^d^			
MMSE total score	27.1 (27.1–27.2)	26.6 (26.5–26.7)	< 0.001
FAB total score	15.3 (15.2–15.4)	15.0 (14.8–15.1)	< 0.001
RBANS total score	85.7 (85.2–86.2)	81.9 (81.0–82.7)	< 0.001
Cognitive dysfunction, *n* (%)	1170 (34.9)	506 (42.0)	< 0.001
RBANS subdomain score			
Immediate memory	90.4 (89.9–91.0)	86.6 (85.7–87.5)	< 0.001
Visuospatial	88.3 (87.7–88.9)	86.5 (85.5–87.5)	0.002
Language	90.5 (90.1–91.0)	88.3 (87.6–89.1)	< 0.001
Attention	88.5 (88.0–89.1)	86.2 (85.3–87.1)	< 0.001
Delayed memory	88.1 (87.5–88.7)	82.4 (81.4–83.3)	< 0.001
Depression (CES-D total score)	3.7 ± 5.9	3.7 ± 5.9	0.989
Anxiety (HADS total score)	1.9 ± 3.1	2.0 ± 3.1	0.628

Data is presented as mean ± SD unless otherwise stated. *P*-values obtained from independent samples *t*-test for continuous data and chi-square test for categorical data. *P* < 0.05 denotes statistical significance

*Abbreviations:* *CES-D* Center for Epidemiological Studies-Depression Scale, *CI* confidence interval, *EGRac* erythrocyte glutathione reductase activation coefficient, *FAB* Frontal Assessment Battery, *HADS* Hospital Anxiety and Depression Scale, *HDL* high-density lipoprotein, *LDL* low-density lipoprotein, *MMSE* Mini-Mental State Examination, *PLP* pyridoxal 5′-phosphate, *RBANS *Repeatable Battery for Neuropsychological Status

^a^*ApoE* ε4 carriers (ε3/ε4 and ε4/ε4)

^b^Age formal education ended

^c^Waist circumference of ≥ 102 cm for males; ≥ 88 cm for females

^d^Displays adjusted means, 95% CIs, controlling for age, sex, education, socioeconomic deprivation, obesity, LDL cholesterol, and depression

**Fig. 2:**
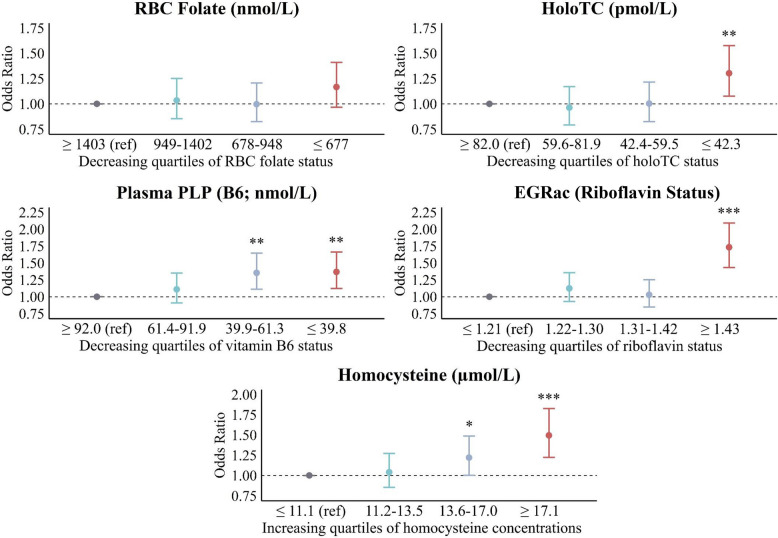
Association of B-vitamin biomarker status with cognitive dysfunction. Data presented as adjusted odds ratios and 95% CIs from logistic regression of cognitive dysfunction (RBANS total score < 80). Biomarkers were categorised into quartiles, with the reference category set at high B-vitamin status. For homocysteine, the reference category equates with high B-vitamin status. Each B-vitamin biomarker was included in separate logistic regression models, adjusted for age, sex, obesity (waist circumference), education, socioeconomic deprivation, and *ApoE* ε4 status (ε3/ε4 and ε4/ε4). Additional adjustments for other covariates, including depressive symptoms, did not materially alter the results.* *p* < 0.05; ** *p* < 0.01; *** *p* < 0.001. Abbreviations: EGRac, erythrocyte glutathione reductase activation coefficient; HoloTC, holotranscobalamin; PLP, pyridoxal 5′-phosphate; RBANS, Repeatable Battery for Neuropsychological Status; RBC, red blood cell; ref, reference category

The association between B-vitamin biomarker status and cognitive dysfunction after adjustment for cognition-related covariates, including *ApoE* status (Fig. 2). Lower holoTC concentrations, the active form of vitamin B12, were associated with a significantly higher risk of cognitive dysfunction (OR_adj_ 1.30; 95% CI: 1.08–1.58; *p* = 0.007). Lower vitamin B6 concentrations (quartiles 3 and 4) were associated with a higher risk of cognitive dysfunction (OR_adj_ 1.35; 95% CI: 1.11–1.65; *p* = 0.002; OR_adj_ 1.37 95% CI: 1.12–1.66; *p* = 0.002, respectively). Poorer riboflavin status (as indicated by higher EGRac values) was strongly associated with cognitive dysfunction, with the highest quartile (indicative of worst riboflavin status) showing a markedly increased risk (OR_adj_ 1.73; 95% CI: 1.44–2.09; *p* < 0.001). Similarly, homocysteine, indicative of low B-vitamin status, was also associated with higher risk of cognitive dysfunction. Conversely, RBC folate and total vitamin B12 were not associated with cognitive dysfunction in this cohort (Additional file 2: Table S1). Plasma homocysteine concentrations showed significant negative correlations with RBC folate, total serum B12, holo-transcobalamin, and vitamin B6. Riboflavin status, assessed by EGRac, was positively correlated with homocysteine (data not shown).

Table 4 presents the interactions between B-vitamins and *ApoE* ε4 on cognitive dysfunction. The *ApoE* ε4 genotype interacted adversely with low holoTC (*β* = 0.336, OR_adj_ 1.40, 95% CI: 1.03–1.90, *p* = 0.030), indicating a 40% increased risk of cognitive dysfunction. There was also an adverse interaction between *ApoE* ε4 status and higher homocysteine, indicative of low B-vitamin status, with a 51% increased risk of cognitive dysfunction (*β* = 0.413, OR_adj_ 1.51, 95% CI: 1.11–2.05, *p* = 0.008). There were no significant interactions observed between the remaining B-vitamins and *ApoE* ε4 on cognitive dysfunction.

**Table 4:** Interactions between B-vitamins and *ApoE* ε4 on cognitive dysfunction in older adults

	**β**	**OR**_**adj**_	**95% CI**	***P***
***ApoE* ε4 x lowest B-vitamin status**				
RBC folate	0.188	1.21	0.89–1.63	0.222
Serum folate	− 0.068	0.93	0.69–1.27	0.662
Total vitamin B12	0.184	1.20	0.89–1.63	0.231
Holotranscobalamin	0.336	1.40	1.03–1.90	0.030
Plasma PLP (vitamin B6)	0.107	1.11	0.82–1.51	0.488
EGRac (riboflavin)	0.041	1.04	0.77–1.41	0.789
Plasma total homocysteine	0.413	1.51	1.11–2.05	0.008

Data presented as standardised *β* coefficients, adjusted odds ratios and 95% CIs.* P*-values were obtained from logistic regression analysis of cognitive dysfunction (RBANS total score < 80). *P* < 0.05 denotes statistical significance. *ApoE* ε4 status (ε3/ε4 and ε4/ε4) and each B-vitamin biomarker were analysed in separate regression models, comparing the lower quartiles (quartiles 1 and 2) to the higher quartiles (quartile 3 and 4) of B-vitamin status. For homocysteine, lower quartiles were used as the reference category, which equates to high B-vitamin status. Each model was adjusted for age, sex, education and socioeconomic deprivation. For further statistical details related to this analysis, please see the Methods section

*Abbreviations:* *CI* confidence interval, *EGRac* erythrocyte glutathione reductase activation coefficient, *OR*_*adj*_ adjusted odds ratio, *PLP* pyridoxal 5′-phosphate, *RBANS* Repeatable Battery for Neuropsychological Status, *RBC* red blood cell

## Discussion

This study investigated the associations of one-carbon metabolism, related B-vitamins and *ApoE* genotype with cognitive function in older adults. The findings, from a large, well-characterised cohort of community-dwelling adults, indicate that lower status of vitamin B12, B6, and riboflavin were each associated with an increased risk of cognitive dysfunction. Also, a novel interaction between *ApoE* and low vitamin B12 as determinants of cognitive dysfunction was identified, suggesting that low B12 status has the potential to modulate the risk of cognitive dysfunction posed by the *ApoE* ε4 genotype. This is one of the first studies to examine the interaction between the *ApoE* genotype with all the B-vitamins involved in one-carbon metabolism in one analysis. Our findings underscore the importance of considering both nutritional and genetic factors when assessing the risk of cognitive dysfunction in ageing populations.

Lower biomarker status of vitamin B12, vitamin B6, and riboflavin were each independently associated with a 30–73% increased risk of cognitive dysfunction, after adjustment for age, sex, obesity, education, socioeconomic deprivation, and *ApoE* ε4 status in this study. Although lower RBC folate status was not associated with cognitive dysfunction, this may be attributed to the generally high folate status among participants in the current study, with only ~ 2% having RBC folate concentrations below the cut-off for deficiency (< 340 nmol/L) [42]. Meta-analyses of observational studies are generally in agreement that lower dietary and more objectively biomarker status of B-vitamins are associated with higher risks of cognitive decline and dementia [43, 44]. In addition, our previous longitudinal study of older adults reported that lower baseline vitamin B6 status (plasma PLP) was associated with a 3.5-fold increased risk of cognitive decline over a 4-year follow-up period [45]. In the current study, higher homocysteine concentrations were associated with a 50% increased risk of cognitive dysfunction, aligning with existing evidence suggesting an association between elevated plasma homocysteine and/or low B-vitamin status with cognitive dysfunction [17, 46, 47]. Consistent with such associations, meta-analyses of intervention studies suggest that B-vitamin supplementation, particularly with folate, B12, and B6, is beneficial for cognitive function and helps to slow the rate of cognitive decline with age [17, 44]. The most robust evidence comes from the 2-year VITACOG RCT which highlighted the efficacy of B-vitamin supplementation in individuals with mild cognitive impairment. Notably, participants with higher baseline homocysteine concentrations (> 11.3 μmol/L) who received B-vitamins experienced a 53% reduction in brain atrophy rates over the 2 years, as measured by MRI [18]. Further analyses from this study [19] demonstrated significant improvements in response to intervention in global cognition, episodic memory, and semantic memory among those with elevated baseline homocysteine (indicative of lower B-vitamin status).

As expected, carriers of the *ApoE* ε4 allele had lower scores on all three of the cognitive assessment tools used in this study, namely, MMSE, FAB and RBANS, and there was a higher prevalence of cognitive dysfunction in the ε4 carriers (42%) compared to non-ε4 carriers (35%). RBANS total scores, as well as scores in the cognitive subdomains—immediate memory, visuospatial, language, attention, and delayed memory—were all significantly lower in ε4 carriers compared to non-ε4 carriers, and the *ApoE* ε4 allele was associated with a 56% increased risk of cognitive dysfunction (based on the RBANS total score < 80). Likewise, the *ApoE* ε4 genotype has been associated with a 15–70% increased risk of cognitive dysfunction in other cohort studies, including a US-based study of individuals of European ancestry [48] and two Chinese cohort studies [49, 50]. The lower risk reported from the China-based studies compared to the current study may be explained by the use in these studies of the MMSE screening tool to assess cognitive function, which has been criticised for its low sensitivity [23]. Overall, the *ApoE* ε4 allele is the best established common genetic risk factor for cognitive dysfunction and AD [7, 51, 52]. A 15-year follow-up study from two cohorts in the UK and France reported that carrying the *ApoE* ε4 allele was associated with an 89% increased risk of dementia compared to non-carriers [53]. Additionally, a single ε4 allele increases the risk of AD threefold compared to the ε3/ε3 genotype, with 40–65% of AD cases carrying this variant [5, 11]. However, not all ε4 carriers develop AD, suggesting that environmental factors may play a role in modulating disease risk [54].

A novel finding of the current study is that *ApoE* ε4 carriers with low B12 status were found to have a higher risk of cognitive dysfunction. Specifically, the combination of *ApoE* ε4 status and low B12 status as measured by holoTC (but not serum total B12) was associated with a 40% increased risk of having cognitive dysfunction after adjustment for relevant covariates. One small study previously reported a significant interaction between serum total B12 and *ApoE* ε4 on cognitive performance in a Chinese population [21]. Similarly, another study in an older Norwegian cohort reported a positive association between serum total B12 and global cognition, specifically among *ApoE* ε4 carriers [55]. The increased risk of cognitive dysfunction observed in the TUDA cohort was further influenced by elevated total homocysteine concentrations (≥ 13.6 µmol/L), which was associated with a 51% increased risk for *ApoE* ε4 carriers. Given that B12 is a key regulator of homocysteine concentrations [56, 57], these findings are particularly concerning as vitamin B12 deficiency is widespread in older adults [57, 58] and an estimated 25% of the global population carries the *ApoE* ε4 allele [59]. Notably, low B12 status in older adults more typically arises as a result of food-bound malabsorption, rather than low dietary intakes (at least in high income countries), and we previously reported that this may be potentially alleviated by consuming fortified foods which provide the crystalline B12 form rather than protein-bound B12 as found in natural food sources [60]. Of note, we have previously reported an important gene-nutrient interaction in this cohort in BMC Medicine [61], in that case with implications for blood pressure and preventing hypertension in genetically at-risk individuals. Taken together, these studies drawing on the data from the well characterised TUDA cohort suggest that there may be important benefits from targeted nutrition strategies for preventing disease in ageing.

In the present analysis, the lowest quartile of holoTC was associated with cognitive dysfunction, whereas this relationship was not evident with total vitamin B12 concentrations. HoloTC (normal range > 40 pmol/L), which represents the biologically active fraction of vitamin B12, is generally considered to be a more sensitive indicator of functional B12 status compared to total vitamin B12 (normal range > 221 pmol/L) which is reported to lack sensitivity and specificity for detecting deficiency [58, 62]. Whereas both folate and vitamin B12 have been widely studied for their relationship with cognitive performance in ageing, the evidence for vitamin B12 is less consistent than for folate [63, 64]. Furthermore, many studies have relied solely on serum total vitamin B12 as a biomarker of status, and although some have demonstrated associations with cognitive outcomes [21, 55], the utilisation of methylmalonic acid or holoTC as biomarkers of B12 status tends to provide stronger support [65–67]. Moreover, lower concentrations of holoTC have been associated with larger volumes of white matter hyperintensities in the brain [68], which are linked with cognition and dementia [69]. In the current study, the lowest holoTC concentrations (< 42 pmol/L) were associated with a significantly increased risk of cognitive dysfunction, whereas no such association was observed when using serum total vitamin B12 as the biomarker of status.

The mechanism by which the *ApoE* genotype increases the risk of AD is not fully understood, but a number of interrelated factors may be implicated. Deficiencies in folate, vitamin B12, and B6 in AD mouse models (Tg2576 and TgCRND8) increase Aβ deposition, likely through hyperhomocysteinemia affecting γ-secretase activity and *presenilin-1* (*Psen1*) promoter methylation. The *Psen1* gene encodes the protein Psen1, a key component of γ-secretase involved in Aβ production. However, supplementation with S-adenosylmethionine, a key methyl donor in one-carbon metabolism, reversed Psen1 overexpression [70]. The ApoE protein plays a crucial role in AD pathogenesis through various mechanisms, including its promotion of amyloid-β (Aβ) accumulation, its influence on the blood–brain barrier, and through neuroinflammatory effects [71]. *ApoE* ε4 accelerates amyloid accumulation, with its effects becoming more pronounced with age. Experimental evidence suggests that *ApoE* isoforms, especially *ApoE* ε4, may modulate Aβ production and clearance, with *ApoE* ε4 being the most detrimental [72]. In addition, *ApoE* ɛ4 carriers exhibit an accelerated breakdown of the blood–brain barrier in the hippocampus and medial temporal lobe, which is linked to cognitive decline independently of Aβ and tau pathology [73]. This breakdown may help explain the lower cognitive scores seen in ɛ4 carriers, particularly as the blood–brain barrier has recently been identified as an early indicator in the clinical stages of AD [74]. Lastly, neuroinflammation is driven by microglia and astrocytes, cells primarily found in the brain and central nervous system, but these cells respond to Aβ accumulation by releasing pro-inflammatory cytokines and engaging in Aβ clearance. However, when microglial and astrocytic activity becomes dysregulated, Aβ phagocytosis can become impaired, leading to persistent inflammation, which may exacerbate AD pathology and contribute to neurodegeneration [72].

This study has several strengths. The TUDA study, with its large, well-characterised cohort, enabled statistical adjustment for a broad range of covariates, which strengthened the validity of the findings. Furthermore, whereas the majority of research to date has used the MMSE as a measure of cognitive dysfunction, the primary outcome in this study was based on the RBANS, a validated, age-adjusted cognitive assessment tool, which provides a reliable and accurate measure of global cognition as well as five cognitive subdomains. Moreover, the inclusion of a comprehensive set of B-vitamin biomarkers (including riboflavin, very rarely measured in human studies) allowed for a much more robust evaluation of B-vitamin status compared to dietary intake measurement and evaluation of their potential roles in cognitive dysfunction than in previous research. However, the study also has limitations. As a cross-sectional study, no causal relationships can be demonstrated, and intervention studies are needed to further explore these findings. This is especially the case in relation to the role of nutritional factors on cognitive outcomes, as poor diet is well recognised both as a cause and consequence of cognitive dysfunction. However, we consider it unlikely that the current findings linking deficiency of specific B-vitamins with cognitive dysfunction are explained simply by poorer dietary intakes in cognitively impaired participants, given that certain biomarkers (e.g. folate) showed no significant relationship.

## Conclusions

In conclusion, this study demonstrated that lower status of vitamin B12, B6, riboflavin, and/or elevated homocysteine concentrations are each associated with a greater risk of cognitive dysfunction, independent of *ApoE* status. The findings also point to a novel gene–nutrient interaction of *ApoE* ε4 genotype with low B12 status (and higher homocysteine) contributing to further risk. Whilst the current findings are based on a cohort of older Irish adults, the results may be generalisable to older populations worldwide and suggest that improving B-vitamin status, either independently or through its modulatory role with the common *ApoE* gene, may have important public health benefits for dementia prevention. Further investigation, ideally in the form of randomised trials, is however required to demonstrate a causative relationship and confirm whether intervention with B-vitamins can confer a beneficial effect in maintaining better cognitive health in at-risk older people.

## Supplementary Information

Additional file 1: Table S1. STROBE Checklist for Cross-Sectional Studies. Outlines how the manuscript complies with the STROBE (Strengthening the Reporting of Observational Studies in Epidemiology) checklist. Each reporting item is listed alongside the corresponding page number in the main manuscript to ensure transparency in reporting.

Additional file 2: Table S1. Association of B-vitamin biomarker status with cognitive dysfunction Description of data: Table S1 presents the associations between individual B-vitamin biomarkers and cognitive dysfunction, expressed as standardised β coefficients, adjusted odds ratios (OR_adj_), and 95% confidence intervals (CIs). The table includes two models: Model 1 adjusted for age, sex, obesity, education, and socioeconomic deprivation; Model 2 additionally adjusted for *ApoE* ε4 status. Biomarkers were categorised into quartiles, and Quartile 1 (high B-vitamin status) served as the reference. A graphical summary of these results is shown in Fig. 2 of the main manuscript.

## Data Availability

The data cannot be shared publicly because of ethical restrictions on sharing sensitive data. Access to the data will be provided following a successful application to the TUDA Consortium. Ulster University’s Research Portal will contain metadata on the dataset and instructions on how to request access to this dataset. The genetic data are currently under embargo.

## Abbreviations

Aβ: Amyloid-β
AD: Alzheimer’s disease
ApoE: Apolipoprotein E
BMI: Body mass index
EGRac: Erythrocyte glutathione reductase activation coefficient
FAB: Frontal Assessment Battery
holoTC: Holotranscobalamin
MMSE: Mini-Mental State Examination
NI: Northern Ireland
OR_adj_: Adjusted odds ratio
PLP: Pyridoxal 5′-phosphate
Psen1: Presenilin-1
RBANS: Repeatable Battery for Assessment of Neuropsychological Status
RBC: Red blood cell
RoI: Republic of Ireland
TUDA: Trinity-Ulster-Department of Agriculture
