# Supplementary material for: Associations of one-carbon metabolism, related B-vitamins and ApoE genotype with cognitive function in older adults: identification of a novel gene-nutrient interaction
Source: BMC Med. 2025 Jul 28;23:440. doi: 10.1186/s12916-025-04276-8 (PMC12302869; doi:10.1186/s12916-025-04276-8)
Supplement: Supplementary file 2 — Additional file 2: Table S1. Association of B-vitamin biomarker status with cognitive dysfunction Description of data: Table S1 presents the associations between individual B-vitamin biomarkers and cognitive dysfunction, expressed as standardised β coefficients, adjusted odds ratios (ORadj), and 95% confidence intervals (CIs). The table includes two models: Model 1 adjusted for age, sex, obesity, education, and socioeconomic deprivation; Model 2 additionally adjusted for ApoE ε4 status. Biomarkers were categorised into quartiles, and Quartile 1 (high B-vitamin status) served as the reference. A graphical summary of these results is shown in Fig. 2 of the main manuscript. [file 12916_2025_4276_MOESM2_ESM.docx]

**Additional file 2**

| **Table S1.** Association of B-vitamin biomarkers status with cognitive dysfunction | | | | | | | | | |
| --- | --- | --- | --- | --- | --- | --- | --- | --- | --- |
|  |  | **Model 1** | |  |  |  | **Model 2** | |  |
|  | **Β** | **OR_adj_** | **(95% CI)** | ***P*** |  | **β** | **OR_adj_** | **(95% CI)** | ***P*** |
| **Red blood cell folate, nmol/L** |  |  |  |  |  |  |  |  |  |
| *Quartile 1*  *≥ 1403 (ref)* | - | - | - | - |  | - | - | - | - |
| *Quartile 2* *949-1402* | 0.031 | 1.032 | (0.852 - 1.249) | 0.750 |  | 0.033 | 1.034 | (0.853 - 1.252) | 0.736 |
| *Quartile 3* *678-948* | -0.010 | 0.990 | (0.819 - 1.197) | 0.916 |  | -0.003 | 0.997 | (0.824 - 1.206) | 0.976 |
| *Quartile 4* *≤ 677* | 0.150 | 1.162 | (0.962 - 1.402) | 0.119 |  | 0.155 | 1.167 | (0.966 - 1.410) | 0.108 |
| **Serum folate, nmol/L** |  |  |  |  |  |  |  |  |  |
| *Quartile 1*  *≥ 44.4 (ref)* | - | - | - | - |  | - | - | - | - |
| *Quartile 2* *25.9-44.3* | 0.034 | 1.035 | (0.852 - 1.258) | 0.729 |  | 0.052 | 1.054 | (0.866 – 1.282) | 0.600 |
| *Quartile 3* *16.4-25.8* | 0.078 | 1.081 | (0.888 – 1.314) | 0.438 |  | 0.089 | 1.093 | (0.897 – 1.330) | 0.378 |
| *Quartile 4* *≤ 16.3* | 0.268 | 1.308 | (1.078 – 1.587) | 0.007 |  | 0.289 | 1.335 | (1.099 – 1.622) | 0.004 |
| **Total Vitamin B12, pmol/L** |  |  |  |  |  |  |  |  |  |
| *Quartile 1*  *≥ 354(ref)* | - | - | - | - |  | - | - | - | - |
| *Quartile 2*  *264-353* | -0.032 | 0.747 | (0.798 - 1.175) | 0.747 |  | -0.029 | 0.971 | (0.800 - 1.180) | 0.769 |
| *Quartile 3* *194-263* | 0.055 | 1.057 | (0.873 - 1.279) | 0.572 |  | 0.070 | 1.073 | (0.885 - 1.300) | 0.473 |
| *Quartile 4* *≤ 193* | -0.021 | 0.980 | (0.809 - 1.186) | 0.833 |  | -0.013 | 0.987 | (0.815 - 1.196) | 0.897 |
| **Holotranscobalamin, pmol/L** |  |  |  |  |  |  |  |  |  |
| *Quartile 1* *≥ 82.0 (ref)* | - | - | - | - |  | - | - | - | - |
| *Quartile 2* *59.6-81.9* | -0.052 | 0.949 | (0.781 - 1.154) | 0.601 |  | -0.038 | 0.963 | (0.791 - 1.171) | 0.704 |
| *Quartile 3* *42.4-59.5* | -0.006 | 0.994 | (0.820 - 1.206) | 0.955 |  | 0.003 | 1.003 | (0.826 - 1.217) | 0.979 |
| *Quartile 4* *≤ 42.3* | 0.260 | 1.297 | (1.072 - 1.569) | 0.008 |  | 0.264 | 1.303 | (1.076 - 1.577) | 0.007 |
| **Pyridoxal 5’-phosphate (vitamin B6), nmol/L** | |  |  |  |  |  |  |  |  |
| *Quartile 1* *≥ 92.0 (ref)* | - | - | - | - |  | - | - | - | - |
| *Quartile 2* *61.4-91.9* | 0.105 | 1.111 | (0.912 - 1.354) | 0.297 |  | 0.103 | 1.109 | (0.909 - 1.352) | 0.309 |
| *Quartile 3* *39.9-61.3* | 0.311 | 1.365 | (1.123 - 1.659) | 0.002 |  | 0.303 | 1.354 | (1.113 - 1.648) | 0.002 |
| *Quartile 4* *≤ 39.8* | 0.329 | 1.389 | (1.143 - 1.689) | < 0.001 |  | 0.313 | 1.368 | (1.124 - 1.664) | 0.002 |
| **EGRac^1^ (riboflavin status)** |  |  |  |  |  |  |  |  |  |
| *Quartile 1*  *≤ 1.21 (ref)* | - | - | - | - |  | - | - | - | - |
| *Quartile 2* *1.22-1.30* | 0.118 | 1.126 | (0.933 - 1.358) | 0.217 |  | 0.117 | 1.124 | (0.931 - 1.357) | 0.223 |
| *Quartile 3* *1.31-1.42* | 0.028 | 1.028 | (0.846 - 1.248) | 0.781 |  | 0.029 | 1.029 | (0.847 - 1.251) | 0.772 |
| *Quartile 4* *≥ 1.43* | 0.556 | 1.743 | (1.445 - 2.103) | < 0.001 |  | 0.549 | 1.732 | (1.435 - 2.091) | < 0.001 |
| **Total homocysteine, µmol/L** |  |  |  |  |  |  |  |  |  |
| *Quartile 1* *≤ 11.1 (ref)* | - | - | - | - |  | - | - | - | - |
| *Quartile 2* *11.2-13.5* | 0.035 | 1.035 | (0.849-1.263) | 0.731 |  | 0.040 | 1.041 | (0.853 - 1.271) | 0.693 |
| *Quartile 3* *13.6-17.0* | 0.190 | 1.210 | (0.993-1.473) | 0.058 |  | 0.200 | 1.221 | (1.002 - 1.488) | 0.048 |
| *Quartile 4* *≥ 17.1* | 0.394 | 1.483 | (1.216-1.811) | < 0.001 |  | 0.402 | 1.495 | (1.224 - 1.826) | < 0.001 |
| Data presented as standardised β coefficients, adjusted odds ratios and 95% CIs. *P*-values were obtained from logistic regression analysis of cognitive dysfunction (total RBANS score < 80). *P* < 0.05 denotes statistical significance. Biomarker status was categorised into quartiles, with the reference category set at high B-vitamin status (Quartile 1). In the case of homocysteine, Quartile 1 (equating with high B-vitamin status) was used as the reference category. Regression models were adjusted for age, sex, obesity, education, socioeconomic deprivation (Model 1), and additionally, *ApoE* ε4 status (ε3/ε4 and ε4/ε4; Model 2). Additional adjustments for other covariates, including depressive symptoms, did not materially alter the results.  ^1^ higher EGRac values are indicative of worse riboflavin status.  Abbreviations: CI, confidence interval; EGRac, erythrocyte glutathione reductase activation coefficient; OR_adj_, adjusted odds ratio; RBANS, Repeatable Battery for Neuropsychological Status; ref, reference category. | | | | | | | | | |
